# Cognitive Functioning When Retiring: Findings From a Swedish Population-Based Longitudinal Study

**DOI:** 10.1093/geroni/igaa057.1498

**Published:** 2020-12-16

**Authors:** Linn Elena Zulka, Isabelle Hansson, Valgeir Thorvaldsson, Linda B Hassing

**Affiliations:** University of Gothenburg, Gothenburg, Sweden

## Abstract

The effects of retirement on cognition are still unclear and empirical evidence is conflicting. Especially for retirement from cognitively demanding jobs, positive as well as negative effects have been reported. Leisure activity engagement has been hypothesized to play an important role in explaining the mixed evidence. In this study, we examine the interplay between job demands before retirement and changes in leisure activities before and after retirement and their relation to post-retirement cognitive functioning. Using data from the HEalth, Aging and Retirement in Sweden (HEARTS) study, cognitive trajectories before and after retirement were modeled in a multi-level piecewise model (N = 2688 observations). Post-retirement memory and reasoning ability were predicted by self-reported work demands and changes in leisure activity engagement. Results imply a stable increase in memory over the retirement transition and less steep increase in abstract reasoning after retirement. Work demands and leisure activity participation were not related to post-retirement cognitive change. Job demands and leisure activity engagement may not play an important role for short-term post-retirement cognitive functioning. These findings support the conclusion that retirement, independent of prior work demands, does not affect cognitive functioning negatively.

